# DNA hydroxymethylation-mediated epigenetic modifications in alcohol use disorder

**DOI:** 10.3389/fnagi.2026.1858336

**Published:** 2026-08-03

**Authors:** Sampath Raghul Kannan, Krishna Santhoshkumar, Ramasamy Tamizhselvi

**Affiliations:** School of Bio Sciences and Technology, Vellore Institute of Technology, Vellore, Tamil Nadu, India

**Keywords:** 5hmC, alcoholic liver disease, AUD, DNA demethylation, ten-eleven translocation, TETs

## Abstract

Alcohol use disorder (AUD) is a chronic neuropsychiatric condition that causes an increased risk of dementia and deaths worldwide. Evidence indicates that alcohol misuse can induce changes in genetic factors. Alcohol-induced DNA epigenetic modification alters brain function and contributes to AUD. Among these modifications, DNA methylation is highly implicated in AUD; however, 5-hydroxymethylation of 5-methylcytosine mediated by ten-eleven translocation (TET) enzymes produces 5-hydroxymethylcytosine (5hmC), which has emerged as a stable epigenetic mark. Recent studies have shown that 5hmC at gene promoters can influence gene expression, especially of genes related to neurotransmitter signaling, alcohol addiction, and cognition. In this review, we summarize findings of TETs-mediated 5-hydroxymethylation in AUD, with special emphasis on the brain and liver. Furthermore, we highlight the key challenges and research gaps in understanding the underlying mechanisms of TETs/5hmC-mediated epigenetic modification in AUD.

## Introduction

Alcohol consumption is common in developing countries, with a significant impact on human health, accounting for more than 200 types of health illnesses. A cycle of binge or excessive drinking, abstinence, and relapse episodes of alcohol use is characterized as alcohol use disorder (AUD) (Koob and Volkow, 2016; Willis et al., 2025). Approximately 10–15% of the global population has AUD, and its clinical implications are heterogeneous, which causes multi-system damage, especially to the liver, gut, and central nervous system (Wei et al., 2023). Furthermore, AUD contributes to the global burden of morbidity and mortality through many physical and psychiatric comorbidities (Le Berre et al., 2017; Rehm, 2011). At the cellular level, alcohol is metabolized into acetaldehyde and then to acetate by alcohol dehydrogenase, catalase, and cytochrome P450 (CYP2E1). Besides these metabolites, reactive oxygen species (ROS) are also produced during the metabolism, which disrupts the redox homeostasis and often induces oxidative stress. Prolonged oxidative stress induced by alcohol use triggers neuroinflammation, cellular apoptosis, and necroptosis, leading to neurodegeneration that contributes to AUD (Kamal et al., 2020; Tsermpini et al., 2022).

Alcoholic liver disorder (ALD) is a continuum of severe liver disease—a name derived termed based on the first stage of ALD, steatosis—characterized by the accumulation of fat in liver parenchymal cells (Kang et al., 2025). Subsequently, steatosis progresses to steatohepatitis as activated immune cells release pro-inflammatory cytokines, leading to substantial hepatocyte death and scarring in the liver. Furthermore, steatohepatitis leads to metabolic dysfunction and activates hepatic stellate cells to release collagen and fibronectin, causing liver fibrosis and liver cirrhosis, which may progress to hepatocellular carcinoma if untreated. This spectrum of ALD is progressive and depends on the extent of alcohol use (Brahadeeswaran et al., 2023; Lai et al., 2024). While the liver is the primary site of alcohol metabolism, its pathological changes and consequences have systemic effects that extend to the brain, underscoring the fact that the liver and brain are interconnected through a bidirectional liver-brain axis. Acetaldehyde and ROS generated in the liver during alcohol metabolism enter systemic circulation and disrupt gut permeability and promote neuroinflammation (Diaz et al., 2025). Conversely, alcohol-induced neuroinflammation can exacerbate peripheral immune activation and liver injury (Mikkelsen et al., 2025). This reciprocal communication highlights the liver–brain axis as a unified pathophysiological framework for AUD. Interestingly, the major risk factors thought to contribute to AUD are not limited to genetic predisposition but also epigenetic modifications. Thus, a clear understanding of the mechanistic association between AUD and epigenetics may further help in the timely identification of liver and brain-metabolic co-morbidities.

Profound reprogramming of gene expression through epigenetics refers to the pattern of chemical changes to the assembly and function of chromatin that regulates the expression of genes without affecting the underlying DNA sequence (Dai et al., 2024). The key epigenetic modifications include methylation and acetylation of histones that pack DNA into chromatin, as well as methylation of DNA itself, which influences the pathophysiological mechanism of AUD. DNA methylation is the addition of a methyl group to the fifth carbon of cytosine in CpG dinucleotides, catalyzed by DNA methyltransferases (DNMTs) that block the transcription and expression of genes. Indeed, the prevailing view was that 5-methylcytosine (5mC) formed by DNA methylation is reversible by restoring the cytosine through other forms of modified cytosine mediated by ten-eleven translocation (TET) enzymes (Dai et al., 2024; López-Moyado et al., 2024). Ample evidence suggests that TETs-mediated 5hmC is involved in the pathophysiological mechanisms underlying AUD. In this review, we summarize the current findings on the contribution of TETs/5hmC to AUD, with a specific focus on the brain and liver. We finally focus on the clinical status of 5hmC as a biomarker of disease stages and a therapeutic target.

## TET enzymes structure and function

**Figure 1:**
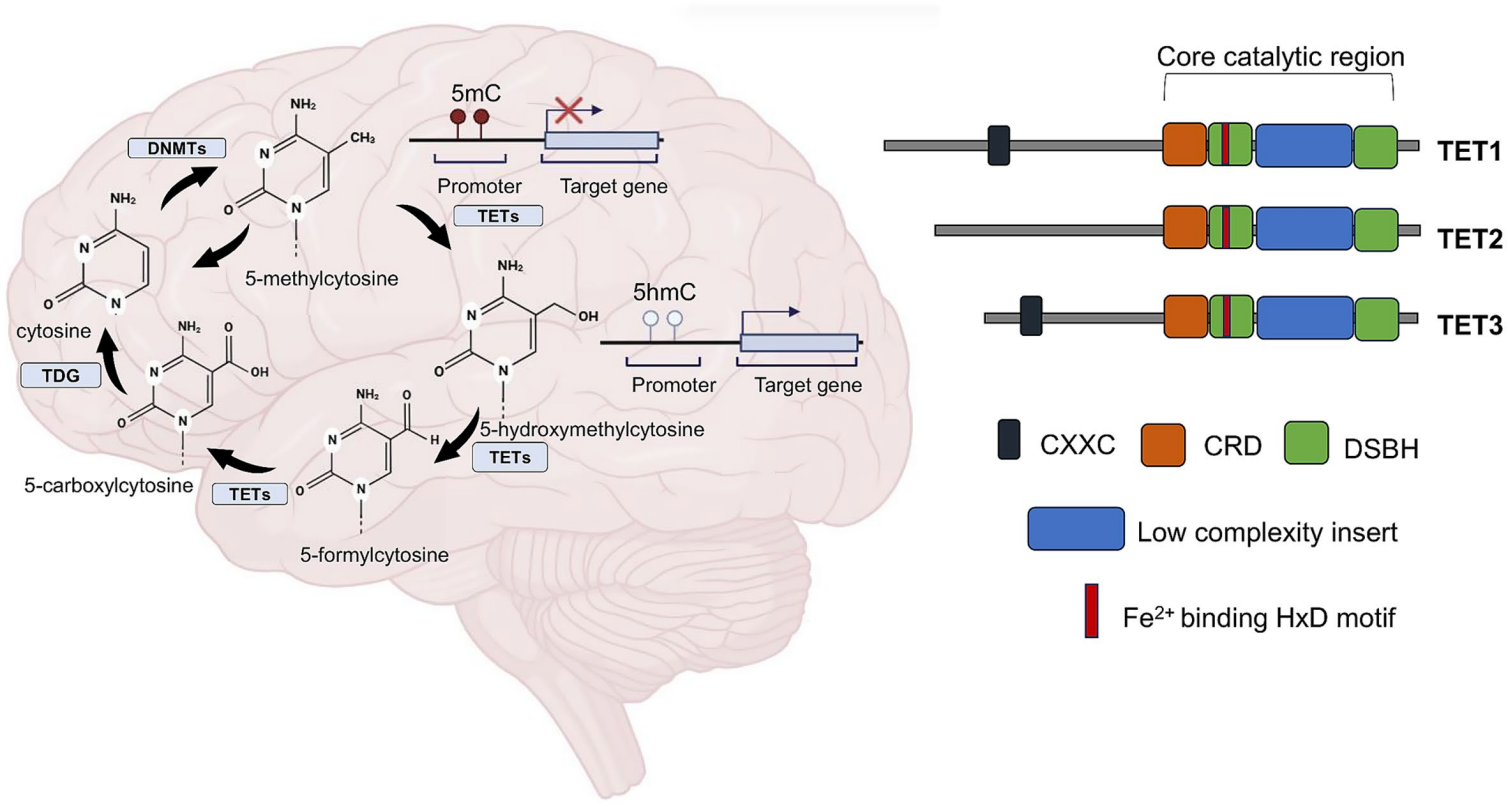
DNA methylation and demethylation pathway. Cytosine is converted to 5-methylcytosine by DNMTs, then to 5-hydroxymethylcytosine, and finally to cytosine by TETs. 5mC in the promoter region of the gene inhibits gene expression, while 5hmC promotes gene expression (left panel). Schematic representation of TET enzymes (right panel). DNMTs, DNA methyltransferases; TETs, ten-eleven translocation; TDG, Thymine DNA glycosylase; 5mC, 5-methylcytosine; 5hmC, 5-hydroxymethylcytosine.

Alongside DNA methylation, which is crucial for cellular processes, DNA demethylation is equally important to balance the gene expression required for cellular homeostasis (Traube and Carell, 2017). Active DNA demethylation is catalyzed by TETs and/or *α*-ketoglutarate-dependent dioxygenases through a series of chemical reactions. Three different TET enzymes, TET1, TET2, and TET3, are found in mammals and have distinctive biological functions (Parker et al., 2019). TETs oxidize 5mC to 5hmC, which is then further oxidized to 5-formylcytosine (5fC) and subsequently 5-carboxycytosine (5caC) (Figure 1). Both 5fC and 5caC are recognized by thymine-DNA glycosylase (TDG), which converts them into 5C (without a methylation mark) by base-excision repair (BER) (Zhang et al., 2023). 5hmC, 5fC, and 5caC are recognized by DNA glycosylases, repair proteins, transcription factors, and chromatin regulators to perform various functions, such as transcriptional regulation, cellular development, and lineage specification (Parker et al., 2019). Among all three intermediates, 5hmC is the most stable and hence the most abundant oxidized form. 5hmC is read by methyl-CpG-binding domain 3 (MBD3), methyl CpG binding protein 2 (MeCP2), and ubiquitin-like with PHD and RING finger domains 2 (UHRF2) to maintain transcriptional programs, cellular identity, and genomes (Kharat and Sharan, 2025).

Structurally, TETs have a catalytic site within the cysteine-rich domain (CRD) located at the carboxy-terminus, and two conserved double-stranded β-helix (DSBH) domains, separated by a low-complexity insert. The CRD provides overall stability during catalysis, while the key residues in the DSBH domain aid in the binding of the co-factors *α*-ketoglutarate and Fe(II), making TETs α-KG/Fe(II)-dependent proteins. TET1 and TET2 have a CpG dinucleotide-binding domain and a CXXC domain at the amino terminal, whereas TET2 lacks this domain due to a chromosomal inversion. TET1/3 preferentially binds to CpG sites in the promoter region, while TET2 binds to gene bodies (Zhang et al., 2023). Importantly, metabolites can modulate this catalytic activity: Vitamin C acts as a cofactor for TET enzymes and promotes DNA demethylation by increasing 5hmC formation (Brabson et al., 2021), whereas 2-HG, succinate, and fumarate are competitive inhibitors of αKG-dependent dioxygenases, including TET, thereby reducing global 5-hmC levels (Arvinden et al., 2017; Lio and Rao, 2019).

Functionally, 5hmC at the promoter region promotes gene activation and maintains gene transcription by protecting from 5mC, while 5hmC within the intragenic region or gene bodies is positively correlated with gene expression (Ehrlich and Ehrlich, 2014; Wen et al., 2014). 5hmC within the introns and exons is a key modification for exon recognition and gene expression alongside 5mC due to the similar binding pattern of MeCP2 to 5hmC and 5mC (Maunakea et al., 2013; Wen et al., 2014). However, the binding affinity of MeCP2 is lower for 5hmC than for 5mC. The binding of MeCP2 to gene promoters may enhance or repress gene expression depending on factors such as phosphorylation, acetylation, and/or 5mC or 5hmC status of the gene (Zhubi et al., 2014).

## TETs-mediated neuroepigenetic modifications in brain development and function

Notably, 5hmC is predominantly found in the brain, and its dynamic regulation is essential for aging, neurodevelopment, and the response to stress (Szulwach et al., 2011; Zhao et al., 2025). The enrichment of 5hmC involves many factors such as reactive oxygen species, vitamin C, and prolonged quiescence (Blaschke et al., 2013; Inoue and Zhang, 2011; Madugundu et al., 2014). Besides, TETs oxidize 5mC to 5hmC more efficiently than they oxidize 5hmC to 5fC and 5caC, which eventually increases the accumulation of 5hmC over time (Occean et al., 2024). Intriguingly, the levels of 5hmC increase sharply during postnatal neurodevelopment, and more specifically, the 5hmC enrichment increases in the genes related to synapse formation, suggesting the importance of 5hmC in early brain development and synaptic functions (Khare et al., 2012; Szulwach et al., 2011).

Loss of TETs in excitatory neurons in postnatal mouse alters the dendrite structures and results in long-term impairments of memory and cognition, which are associated with the impaired synaptic plasticity in the absence of TETs activity (Liu et al., 2024). Furthermore, loss of *Tet1* impairs memory and knockdown of *Tet3* reduces synaptic plasticity in glutamatergic neurons, highlighting the importance of TETs in brain development (Rudenko et al., 2013; Yu et al., 2015). Besides their role in early brain development, 5hmC has a significant impact on neurogenesis and cognition in adults (Antunes et al., 2021; Greer et al., 2021). In contrast to the postnatal brain, loss of TETs improves cognition in adults. In particular, knockdown of *Tet2* positively regulates synaptic gene expression and enhances memory in the adult mice (Pratt et al., 2022). Recently, *Tet2* has been associated with Alzheimer’s disease by inducing inflammation and with the pathogenesis of Parkinson’s disease, indicating that the activity of TETs is cell type-specific and context-dependent (Carrillo-Jimenez et al., 2019; Marshall et al., 2020; Zhao et al., 2025).

In addition to these developmental and structural roles, 5hmC is dynamically regulated by neuronal activity, driving the activity-dependent transcription of immediate-early genes that couple synaptic stimulation to long-term plasticity. Loss of *Tet1* is associated with the downregulation of several neuronal-activity-regulated genes, such as *Npas4*, c-*Fos*, and *Arc*, along with altered synaptic plasticity (Rudenko et al., 2013). Importantly, dysregulation of activity-dependent transcription mediated by 5hmC modification has been implicated in maladaptive neuroadaptations by affecting synaptic plasticity and learning and memory, providing a mechanistic basis for addiction-related plasticity in AUD (Dick et al., 2021).

## DNA modifications in AUD pathophysiology

Alcohol induces DNA methylation and affects gene regulation and function, leading to AUD. In particular, alcohol-induced DNA methylation increases excessive alcohol-seeking and drinking behaviors in animals and humans, contributing to AUD (Gao et al., 2026; Gavin et al., 2016). Alcohol differentially regulates the expression of *DNMTs* and the enzymatic activity of DNMTs in the brain, liver, and pancreas (Ajoolabady et al., 2022; Jarczak et al., 2023; Routh and Manickam, 2024). DNA methylation is mediated by three enzymes in mammals: DNMT1, DNMT3a, and DNMT3b. Acute alcohol exposure decreased DNMT activity, while *DNMT1* and *DNMT3a* were found to be upregulated in the brain of adolescent rats (Sakharkar et al., 2014). In an intermittent alcohol exposure paradigm, DNMT activity and expression of DNMT1 and DNMT3b were increased, which was also reflected in the DNA methylation of genes in adolescent rats (Sakharkar et al., 2019). Chronic exposure for 3 weeks in alcohol dependent rats increased *DNMT1* expression and 5mC levels in the brain (Barbier et al., 2015). Genome-wide association studies of the human brain revealed methylation genes and the associated pathways differentially (Andrade-Brito et al., 2024; Clark et al., 2022; Lohoff et al., 2022). However, minimal overlapping findings were found among the findings of these studies, highlighting the complex mechanism and the region-specific effects of alcohol on the AUD brain.

Emerging studies revealed alcohol use alters the TETs expression and 5hmC levels in the brain, liver, and blood (Andrade-Brito et al., 2024; Ji et al., 2019; Koller et al., 2019). Until recently, 5hmC was overshadowed by 5mC, as it could not be distinguished by conventional bisulfite sequencing. Recent technologies, such as reduced representation oxidative bisulfite-sequencing (RRoxBS), use potassium perruthenate, which exclusively oxidizes 5hmC to 5fC, after which 5fC is subsequently converted to uracil by bisulfite treatment (Zhang et al., 2023). Studies focusing on DNA hydroxymethylation in alcohol use are limited and have been conducted in the brain, liver, and blood of both animals and individuals with AUD. In the following sections, we summarize the findings on TETs and 5hmC in AUD brain and liver.

## 5hmC-mediated neuroepigenetic modification in AUD brain

In a recent study, 5hmC was investigated in the human orbitofrontal cortex (OFC) using RRoxBS, which identified 363 genome-wide significant CpG sites associated with AUD based on 5hmC, including 213 hypomethylated and 150 hypermethylated CpG sites. Among them, 66% of the genome-wide CpG sites based on 5hmC were located at promoters, and 17%were found within intragenic regions. Furthermore, 11% and 6% of 5hmC were found within introns and exons, respectively (Andrade-Brito et al., 2024). *GAD1*, *DLX1*, and *DLX2* are some of the genes with differential 5hmC that were identified to be associated with AUD (Andrade-Brito et al., 2024). All three genes are hyperhydroxymethylated in individuals with AUD and are connected to the GABAergic neurotransmission system. *GAD1* is a well-known gene encoding the enzyme glutamate decarboxylase, which converts glutamate to *γ*-aminobutyric acid (GABA). Hyper-5hmC at the *GAD1* could potentially upregulate its gene expression, which has been widely observed in individuals with AUD. The change in the enrichment of 5hmC could be a compensatory mechanism to increase the reduced levels of GABA often observed in AUD (Edenberg et al., 2010; Hade et al., 2021; Underwood et al., 2019). DLX1 and DLX2 are almost exclusively expressed in neurons, where they regulate the differentiation of GABAergic neurons and GABA levels in the brain by activating the transcription of *Gad* isoforms (Le et al., 2017; Pla et al., 2018). Hyper-5hmC found in the exon region of *DLX1* and *DLX2* is associated with increased gene expression, which is crucial for neuronal differentiation. The activity of DLX1/2 is essential during the early developmental phase, correlating with high 5hmC enrichment; however, its function in postnatal brain development remains poorly characterized. In a brainstem stroke model, Dlx2, along with BDNF, promoted the differentiation of neural stem cells and replaced the lost GABAergic neurons, indicating that DLXs could function through the regeneration and recovery of neurons in the adult brain (Tang et al., 2023). Therefore, we hypothesize that regulation of the enrichment of 5hmC on *DLX1/2* could be a recovery or regulatory mechanism to replace or regenerate the neuronal loss observed in alcohol-induced neurodegeneration. However, there is no direct evidence of *DLX1/2* differential regulation and its role in the pathogenesis of AUD. Collectively, hyper-5hmC enrichment in GAD1, DLX1, and DLX2 may represent an adaptive epigenetic response aimed at restoring inhibitory balance through enhanced GABAergic differentiation and neurotransmission. These changes could influence corticolimbic circuit excitability and contribute to the neuroplastic adaptations underlying alcohol reinforcement and dependence.

Another gene, GABA type A receptor subunit delta (GABRD), is an inhibitory receptor involved in GABAergic signaling that mediates the reward circuitry in alcohol abuse; it is hypermethylated at its promoter region with subsequent downregulation of its expression in the human cerebellum (Gatta et al., 2017; Granchi et al., 2026). Notably, the absence of significant changes in 5hmC levels, together with reduced TET1 expression, suggests an impaired demethylation pathway leading to GABRD hypermethylation, even without marked alterations in DNMT expression (Gatta et al., 2017). Knockdown of GABRD in the medial nucleus accumbens (NAc) shell has been associated with reduced alcohol drinking, which may be due to decreased tonic inhibition, thereby potentially suppressing neuronal excitability that is crucial for alcohol reinforcement (Mihalek et al., 2001; Nie et al., 2011).

Increased expression of *GAD1*, *DLX1*, and *DLX2* could increase the differentiation of GABAergic neurons and GABA levels in the brain, eventually increasing the inhibitory neurotransmission. Intriguingly, inhibitory and excitatory neurons are vulnerable to alcohol-induced effects in glutamatergic and GABAergic signaling, which influence dendritic plasticity along with that of glial cells, which induces neuronal firing in AUD (Huggett et al., 2026; Warden et al., 2026). Prolonged inhibitory signaling can increase neuronal hyperexcitability, thereby promoting alcohol reinforcement and dependence (Dharavath et al., 2023; Fish and Joffe, 2022). In contrast, reduced expression of GABRD is associated with reduced alcohol drinking. Together, reduced expression of GABRD decreases the availability of GABA receptors despite the increased GABA levels, which could weaken tonic inhibition and disrupt GABAergic networks. This may contribute to disrupted inhibitory tone across corticolimbic circuits, potentially influencing alcohol reinforcement, dependence, and relapse vulnerability.

*GATA4* encodes a transcription factor involved in drug addiction and is a crucial regulator of astrocyte proliferation, while its loss leads to malignancy in the brain (Agnihotri et al., 2011). *GATA4 i*s hypomethylated and hypo-hydroxymethylated at the promoter CpG sites. Hypo-5hmC at the promoter CpG site is associated with activating gene expression by reducing the repressive influence of 5mC, thereby facilitating transcription factor binding and promoting an open chromatin state (Andrade-Brito et al., 2024; Ehrlich and Ehrlich, 2014). This supports the upregulation of *GATA4* observed after high alcohol exposure (Zhong et al., 2010). Several genetic variants of GATA4 have been identified to contribute to the pathogenesis of AUD (Karpyak et al., 2014; Mauro et al., 2018). The hypomethylation and hypo-hydroxymethylation of *GATA4* promoter CpGs, associated with its upregulation under high alcohol exposure, indicate activation of astrocytic and transcriptional pathways that may support neuroinflammatory responses. These epigenetic changes could integrate glial remodeling into the broader pathophysiological framework of AUD.

Among the neurotransmitter systems affected by alcohol use, the endogenous opioid system (EOS) is important in the development of alcohol and drug addictions. The kappa (*κ*) opioid receptor (KOR) is one of the receptors in the EOS to which dynorphins (DYN) bind to it, and the DYN/KOR system regulates mood and motivation (Logrip et al., 2008; Wee and Koob, 2010). Repeated alcohol use activates the system and reinforces excessive alcohol seeking and consumption during the withdrawal phase (Lindholm et al., 2001; Logrip et al., 2008; Sirohi et al., 2012). Meanwhile, the use of a KOR antagonist specifically in the amygdala reduced the self-administration of alcohol and alcohol reinforcement during the withdrawal phase (Kissler and Walker, 2016). Several epigenetic modifications were reported to mediate *Kor* expression and the DYN/KOR system (Siciliano et al., 2015; Sirohi et al., 2012). Recently, it was demonstrated that increased *Kor* expression was associated with increased 5hmC at the *Kor* promoter, while 5mC remained unaltered in the NAc of alcohol preferring Alko Alcohol (AA) rats exposed to intermittent alcohol drinking (Niinep et al., 2021). In the same study, global 5mC and 5hmC were elevated, while no significant changes were found in the expression of TETs and their enzyme activity in the alcohol preferring AA rats exposed to intermittent alcohol drinking. This increase in 5hmC levels, even in the absence of changes in TET activity, could possibly be due to the increased levels of 5mC, as a compensatory mechanism to maintain DNA methylation/demethylation balance, indicating that the 5mC/5hmC enrichment is tightly regulated and the underlying mechanism is unclear. The aberrant DNA methylation/demethylation process observed in alcohol preferring rats may reflect not only the effect of alcohol exposure but also the underlying genetic predisposition to alcohol preference (Niinep et al., 2021).

**Figure 2:**
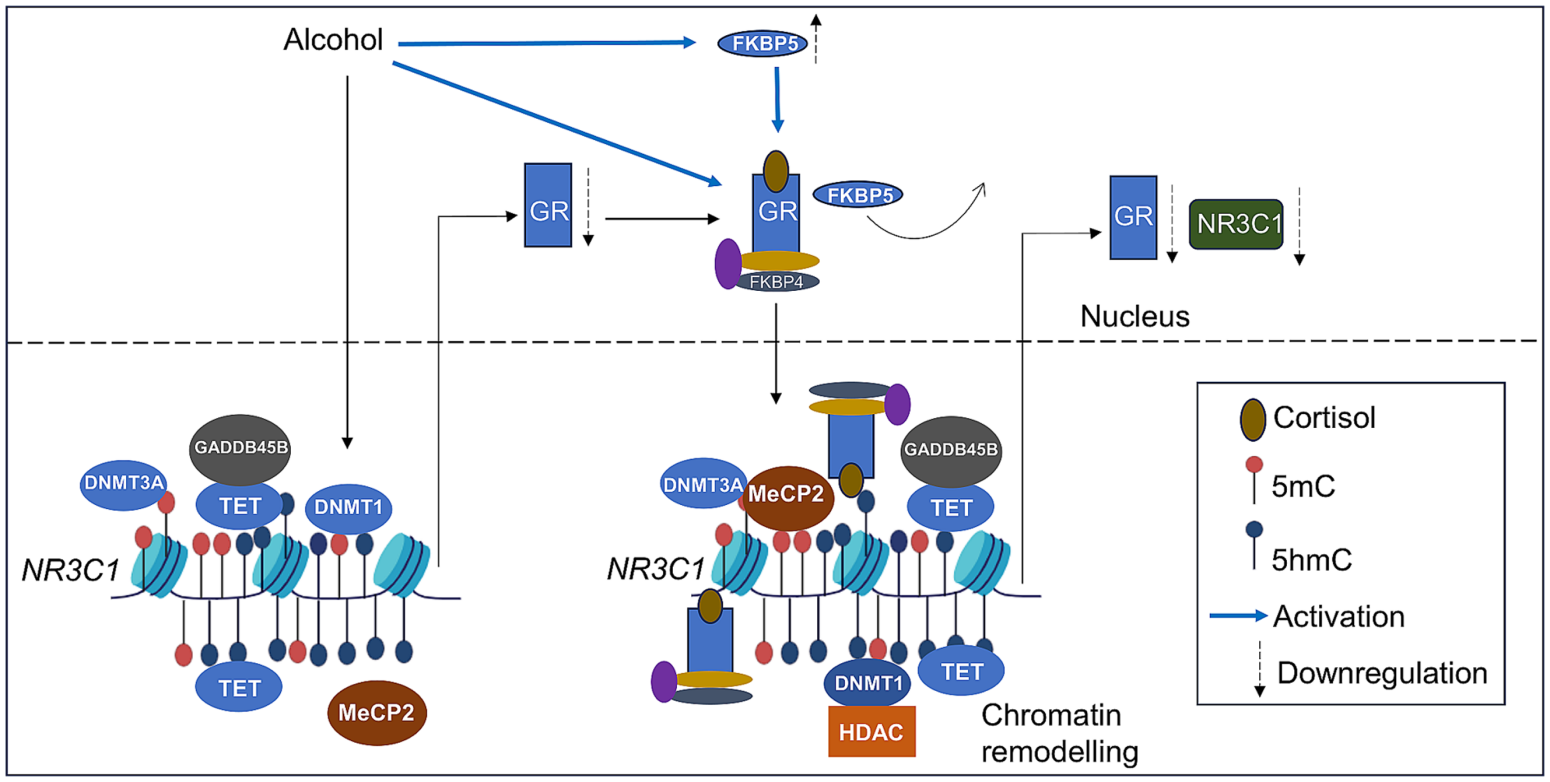
Alcohol-induced epigenetic modification of glucocorticoid signaling. Alcohol increases 5mC and 5hmC in the NR3C1 gene and downregulates its gene expression, resulting in the reduced levels of glucocorticoid receptor (GR), while showing reduced binding of 5hmC toward MeCP2. Meanwhile, alcohol activates GR signaling and FKBP5 and increases the expression of FKBP5. Activated FKBP5 dissociates from the GR complex, and the GR complex translocates to the nucleus, binds to the *NR3C1* gene, and recruits MeCP2, DNMTs, TETs, and other nuclear remodeling factors to repress gene expression, resulting in decreased NR3C1 and GR. NR3C1, nuclear receptor subfamily 3 group C member 1; FKBP5, FK506-binding protein 51; 5mC, 5-methylcytosine; 5hmC, 5-hydroxymethylcytosine.

Nuclear receptor subfamily 3 group C member 1 (NR3C1) encodes the glucocorticoid receptor (GR) involved in the stress response pathway and AUD pathogenesis. Upon translocating to the nucleus, these receptors act as transcriptional factors for glucocorticoid-responsive genes and *NR3C1* itself. Hypermethylation with higher 5hmC over 5mC has been found in *NR3C1* exons along with reduced gene expression of *NR3C1* in the prefrontal cortex (BA10) of human donors with AUD (Gatta et al., 2021). Reduced expression of *DNMT* and *TET* prevents the conversion of 5hmC to 5caC, causing accumulation of 5hmC, which alters DNA methylation dynamics and reduces MeCP2 binding at the promoter, contributing to *NR3C1* downregulation (Ben-Shachar et al., 2009; Chahrour et al., 2008). The reduction in the binding of 5hmC to MeCP2 could be due to the lower binding affinity of 5hmC than that of 5mC. Alternatively, alcohol activates NR3C1 and induces FKBP5, a negative regulator of *NR3C1*, thereby promoting the translocation of NR3C1 into the nucleus (Binder, 2009; Cruz et al., 2023). Once inside the nucleus, NR3C1 recruits MeCP2 and DNMT-containing chromatin remodeling complex to modify chromatin accessibility and reinforce the repression of *NR3C1* (Gatta et al., 2021; Zannas and Chrousos, 2017) (Figure 2). This dysregulation of DNA methylation dynamics is associated with an imbalance in the 5hmC/5mC levels, which alters stress-responsive genes and disrupts the GR signaling and hypothalamic–pituitary–adrenal (HPA) axis in AUD (Gatta et al., 2021). Disruption of GR signaling has a profound impact on alcohol dependence and withdrawal symptoms by impairing synaptic plasticity in corticolimbic circuits, altering glutamatergic and dopaminergic transmission, and increasing relapse vulnerability (Farokhnia et al., 2022; Friske et al., 2025; Khom et al., 2022). An altered HPA axis and GR signaling due to an imbalance in 5hmC/5mC may likely contribute to maladaptive plasticity in the PFC, increasing the risk of alcohol relapse vulnerability. GADD45b, involved in demethylation, is implicated in alcohol-drinking behavior. Acute ethanol injection increased both *Bdnf* and *Gadd45b* expression, indicating a positive correlation of *Gadd45b* with *Bdnf* expression. Furthermore, *Gadd45* +/− mice consume more alcohol, suggesting the importance of demethylation on *Bdnf* and altered drinking behavior (Gavin et al., 2016). Besides, Gatta et al. (2017, 2021) showed that *GADD45b* is upregulated in the prefrontal cortex and the cerebellum of human donors with AUD but did not explain the significance of GADD45b in gene expression. Acute exposure to alcohol in adolescent mice increased the expression of *Gadd45* isoforms but found no significant changes in TET1 and 5hmC at the *Npy* and *Bdnf* promoters in the amygdala (Sakharkar et al., 2019). In another study, *Tet1* expression was upregulated in the NAc following chronic intermittent ethanol vapor exposure but did not study DNA methylation (Finegersh et al., 2015).

A comprehensive study of 5mC and 5hmC in the brain revealed 5hmC in BAR/IMD domain-containing adaptor protein 2 (*BAIAP2*), which was associated with AUD in the brain (Clark et al., 2022). *BAIAP2* is responsible for cognitive function, and its aberration or lack of *BAIAP2* is implicated in many psychiatric disorders (Kang et al., 2016; McKinney et al., 2017). One study reported altered expression of *BAIAP2* in the fetal alcohol exposure model (Lussier et al., 2015). It could be hypothesized that alcohol use alters *BAIAP2* expression, leading to cognitive changes in AUD patients. However, further studies are required to validate the role of BAIAP2 in AUD.

**Figure 3:**
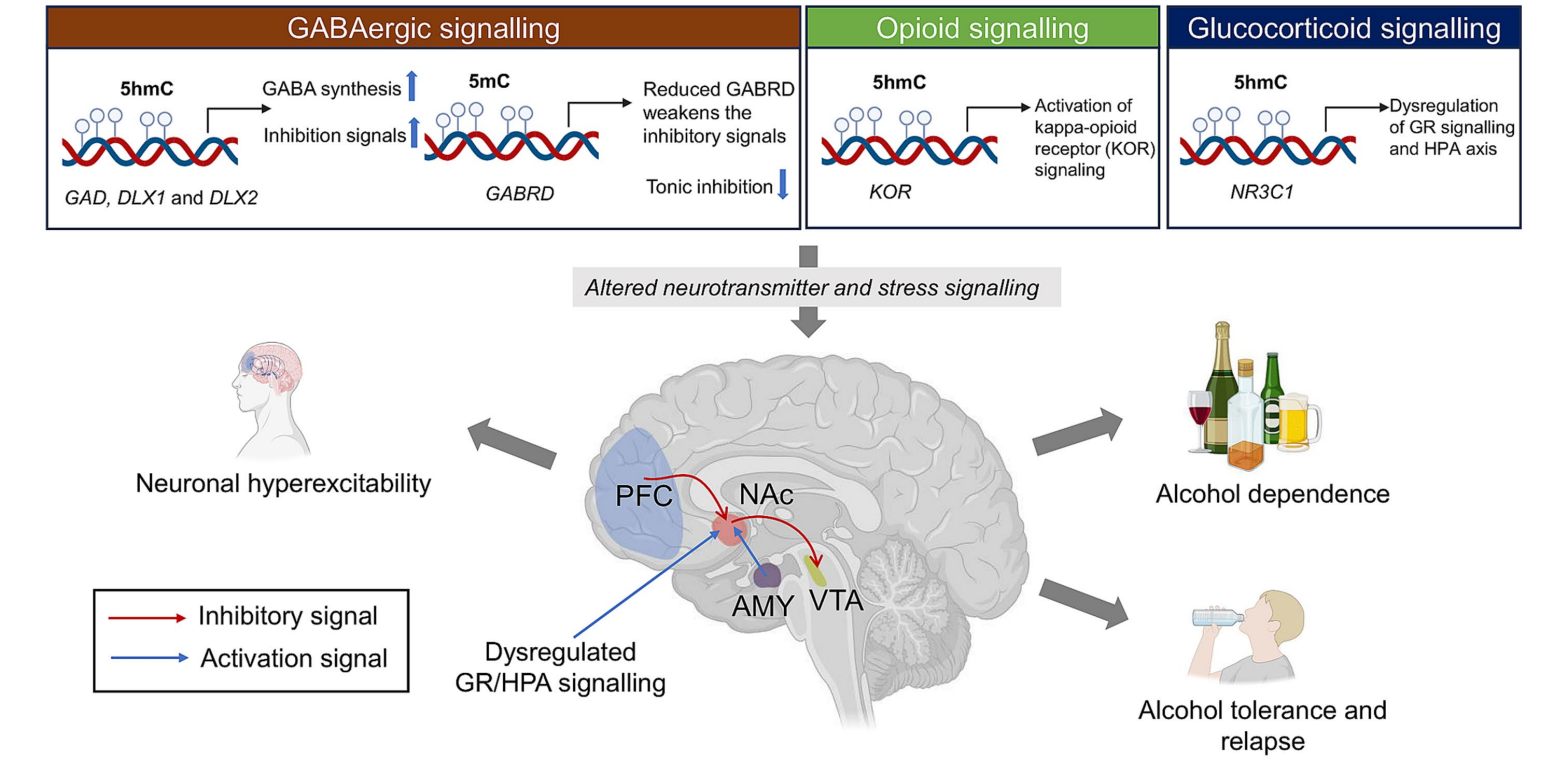
Alcohol-induced hydroxymethylation in the brain. Increased levels of 5hmC in GAD, *DLX1* and *DLX2* could increase the differentiation of GABAergic neurons and GABA synthesis, which eventually increases inhibitory neurotransmission, while an imbalance in 5mC/5hmC with increased 5mC in GABRD reduces receptors, which weakens inhibitory signals despite increased inhibitory signals from GABA. Furthermore, increased 5hmC in the kappa-opioid receptor (*KOR*) activates KOR signaling, and increased 5hmC in *NR3C1* dysregulates GR and HPA signaling. Altered inhibitory neurotransmission from the PFC triggers VTA through NAc, leading to neuronal hyperexcitability and alcohol dependence, while opioid signaling from AMY activates NAc, leading to alcohol reinforcement-related behaviors. *NR3C1*, nuclear receptor subfamily 3 group C member 1; PFC, prefrontal cortex; NAc, nucleus accumbens; AMY, amygdala; VTA, ventral tegmental area; 5mC, 5-methylcytosine; 5hmC, 5-hydroxymethylcytosine.

Furthermore, gene enrichment analysis by Brito et al. identified ‘homophilic cell adhesion via plasma membrane adhesion molecules’, ‘neurogenesis’, ‘embryonic organ development’, ‘Wnt signaling’, ‘calcium ion transport’ and ‘developmental process’ as significantly enriched gene ontology pathways for differential 5hmC genes (Andrade-Brito et al., 2024). Previous studies have collectively identified a minimal number of genes with 5hmC in AUD brain compared to 5mC. Genes involved in the neurotransmitter system, such as *GAD1*, *DLX1*, *DLX2*, *GABRD*, and *KOR*; receptor-associated genes, such as *NR3C1;* and the cognitive function gene *BAIAP2* are identified as differentially hydroxymethylated in the brain of animals or donors with AUD (Table 1). Taken together, these gene-specific enrichments of 5hmC converge on a mechanistic framework, in which differential enrichment or disrupted 5hmC/TET-mediated regulation affects GABAergic, glutamatergic, and opioid signaling networks. Enhancement of GABA production through hyper-5hmC in GAD1, DLX1, and DLX2 may represent compensatory upregulation of inhibitory neurotransmission, whereas hypermethylation of GABRD without 5hmC involvement disrupts tonic inhibition. In parallel, aberrant hydroxymethylation and downregulation of NR3C1 impair glucocorticoid receptor signaling and hypothalamic–pituitary–adrenal (HPA) axis regulation, thereby weakening stress adaptation and increasing relapse vulnerability. Moreover, alcohol-induced activation of the dynorphin/*κ*-opioid receptor (DYN/KOR) system mediated by hyper-5hmC of *KOR* further reinforces excessive alcohol seeking and consumption, particularly during withdrawal. The impaired inhibitory neurotransmission affects the PFC-OFC-NAc interconnected corticolimbic network that integrates reward valuation, inhibitory control, and decision-making. GABAergic signals from the PFC and OFC converge onto NAc, modulating activity in the ventral tegmental area (VTA) and reshaping reinforcement effects (Figure 3). Epigenetic alterations induced by alcohol on genes related to GABAergic signaling within the interconnected network impair the inhibitory tone and excitatory drive, weakening PFC-mediated top-down control while amplifying OFC-driven reward bias. This imbalance in circuit connectivity contributes to maladaptive plasticity, manifesting as compulsive alcohol seeking, impaired cognitive control, and heightened relapse vulnerability.

**Table 1:** List of genes with 5hmC in AUD brain.

Gene with 5hmC	5hmC levels compared to control	Method to study 5hmC	Gene function	Reference
*GAD1*	Increased	Reduced representation oxidative bisulfite-sequencing (RRoxBS)	GABA synthesis from glutamate	Andrade-Brito et al. (2024)
*DLX1*	Increased	RRoxBS	GABAergic signaling	Andrade-Brito et al. (2024)
*DLX2*	Increased	RRoxBS	GABAergic signaling	Andrade-Brito et al. (2024)
*GATA4*	Decreased	RRoxBS	Involved in drug addiction	Andrade-Brito et al. (2024)
KOR	Increased	Real time-PCR with glycosylated site-specific primers	Involved in alcohol seeking and drinking	Niinep et al. (2021)
*NR3C1_1H_*	Increased	Hydroxymethyl-DNA-immunoprecipitation (hMeDIP)	Involved in the stress response pathway	Gatta et al. (2021)
*BAIAP2*	Increased	5hmC Selective Chemical Labeling Sequencing (hMe-Seal)	Responsible for cognitive function	Clark et al. (2022)

Nonetheless, most of the genome-wide epigenetics studies on 5hmC have been focused only on the bulk brain tissue or specific regions, masking the cell-type-specific 5hmC enrichment. Investigations into 5hmC-mediated epigenetic modifications are gaining attention in AUD because of its abundance in the brain and its impact on the development of AUD pathogenesis. Spatial single-cell epigenomics, together with transcriptomes and functional outcomes, in genetically manipulated animal models would provide much deeper insights into the significance of 5hmC enrichment in specific regions or cell types during alcohol dependence, alcohol withdrawal, and alcohol reinforcement behaviors.

## TET enzymes in epigenetic remodeling of ALD

**Figure 4:**
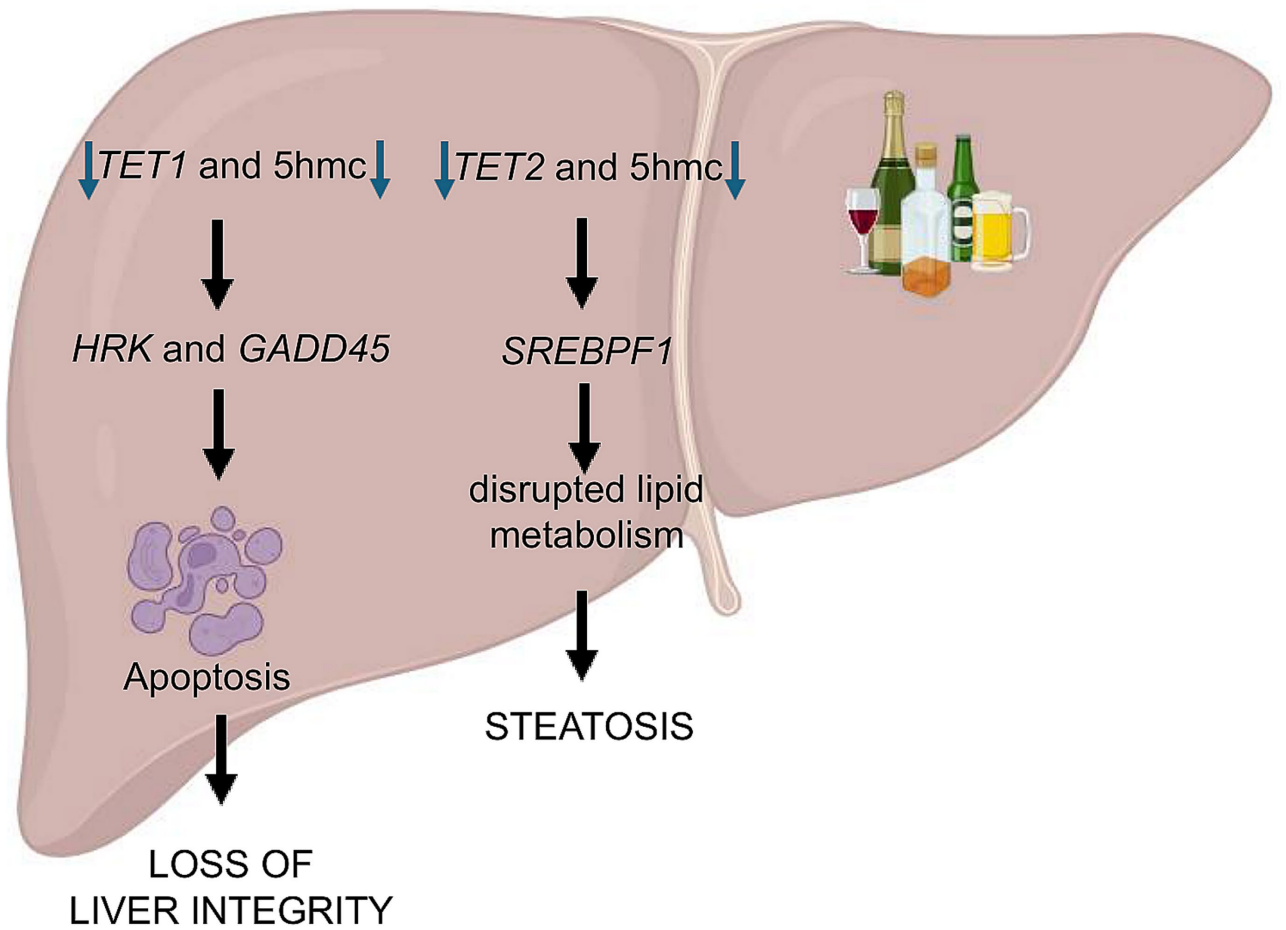
Alcohol-induced changes in TETs/5-hydroxymethylation in alcoholic liver disease (ALD). Reduced expression of TET1 and 5hmC in ALD induces apoptosis of hepatocytes through HRK and GADD45, while reduced expression of TET2 and 5hmC leads to steatosis.

Recently, the precise mechanism of TET enzymes in the pathogenesis of ALD has gained considerable importance. Previous studies have revealed a sharp contrast between how DNA methylation and demethylation mechanisms alter gene expression in ALD. Alcohol intake reduces the expression of *TET1* and 5-hmC levels, whereas the levels of *TET2* and *TET3* remain unchanged (Ji et al., 2019). The TET enzymes (TET1, 2 and 3) are key epigenetic regulators that oxidize 5mc to 5hmc, thereby erasing the methylation in the DNA and helping to dynamically modulate (activate) gene expression (Zhang et al., 2023). TET-induced changes may affect key liver pathways in ALD, such as cell death and fibrosis, lipid metabolism, and inflammation. Reduced expression of TET1 and decreased 5hmC contribute to hepatocyte apoptosis in individuals with ALD by increasing the expression of DNA damage markers HRK and GADD45α, eventually damaging liver integrity (Ji et al., 2019). In hepatocellular carcinoma (HCC) donors and tumor models, alcohol exposure significantly decreased TET2 gene and protein expression, while TET2 was negatively correlated with the survival of HCC patients (Figure 4). Reduced expression of TET2 under alcohol exposure could promote stemness, metastasis, and tumor growth in HCC (Chen et al., 2021). However, 5hmC levels in the HCC human donor tissue or tumor model were not investigated. Intriguingly, a study investigated whether iron supplementation alters global 5hmC in the livers of alcohol-exposed rats, given that TETs are iron-dependent enzymes. The findings revealed that ethanol decreased the 5hmC levels, but no difference was found in iron-supplemented rats (Tammen et al., 2016).

Besides the role of TETs in gene transcription by converting 5mC to 5hmC, TET2 is also reported to influence demethylation of mRNA post-transcriptionally and affect its function (Delatte et al., 2016). TET2 modulates the nuclear localization of sterol regulatory element binding protein 1 (SREBP1) mRNA by interacting with and demethylating its 3’UTR. Consequently, this facilitates the nuclear retention of *Srebp1* and affects its protein expression, leading to disruption in lipid metabolism, which progresses to fatty liver and steatosis (Li et al., 2024). Furthermore, TET/5hmC is also implicated in non-alcoholic fatty liver, hepatocellular carcinoma, liver fibrosis, and liver cirrhosis (Feng et al., 2023; Pirola et al., 2015; Qiu et al., 2023). Together, studies did not exclusively focus on 5hmC in genome-wide or in any particular gene in ALD. Mostly, expression of TETs, global 5hmC levels, and genetic manipulation of TETs have been studied in the liver of individuals with AUD or animal models (Table 2). This could be due to the lower abundance of 5hmC in the peripheral organs compared to the brain. However, the lack of sufficient research limits our understanding of 5hmC-mediated epigenetic modification and the balance of DNA methylation and demethylation in ALD.

**Table 2:** List of genes with 5hmC in ALD.

Gene with 5hmC	5hmC levels compared to control	Method to study 5hmC	Gene function	Reference
*GADD45*α	Increased	Real-time PCR	Stress sensors that regulate cell cycle checkpoints (G2/M), DNA repair (NER/BER), and apoptosis. Promotes DNA damage in ALD.	Ji et al. (2019)
HRK	Increased	Real-time PCR	Triggers programmed cell death by binding and inhibiting anti-apoptotic proteins. Promotes hepatocyte apoptosis and DNA damage in ALD	Ji et al. (2019)
Srebp1	Increased	qPCR and Western blot	SREBP1 protein is a key regulator of hepatic lipid synthesis	Li et al. (2024)
Fkbp5	Increased	Immunoblotting and co-immunoprecipitation	Co-chaperone protein that interacts with heat shock protein 90 (HSP90) and glucocorticoid receptor (GR) complex.	Kusumanchi et al. (2021)

## Limitations and future directions

Advancements in high-throughput sequencing technologies such as RRoxBS, hMe-Seal, and nanopore sequencing have greatly expanded our understanding of 5hmC modifications mediated by TET enzymes over the past decade. However, studies of TET-mediated 5hmC in AUD remain in their infancy. Only a few epigenomic investigations have reported differential enrichment of 5hmC in the brains of individuals with AUD, and the complete landscape of 5hmC in human brain donors remains incompletely characterized. Given the complexity of the brain and the disproportionately high enrichment of 5hmC in neuronal populations, it is increasingly evident that 5hmC patterns are likely region-specific and cell-type-specific. Current findings are limited due to (1) by relying on bulk brain tissue, which obscures cell-type specific enrichment, (2) the lack of integrative epigenomic-transcriptomic approaches, which restricts insight into the functional consequences of gene-specific 5hmC enrichment, (3) the lack of genetic manipulation studies in animal models to validate behavioral outcomes, (4) small cohorts with different demographic characteristics, and (5) the lack of locus-specific epigenetic editing tools.

Future studies should use multi-omics analyses to investigate 5hmC in individuals with AUD and elucidate the role of 5hmC-mediated epigenetic modification in AUD. Comprehensive mapping of the 5-hydroxymethylome across individual cell-type populations, spatially resolved brain regions, and activity-dependent transcriptional programs will be essential to uncover the functional consequences of 5hmC dysregulation. Such integrative approaches, combined with genetic manipulation in animal models, will provide mechanistic insights into how 5hmC contributes to alcohol dependence, withdrawal, and relapse behavior. Furthermore, modulators such as vitamin C, which can enhance TET activity, and gene locus-specific epigenome editing tools would contribute to the development of therapeutic strategies for AUD in the context of DNA demethylation.

## Conclusion

TET-mediated 5hmC is a recognized stable DNA epigenetic modification. However, until recently, studies mostly focused on 5mC as the primary epigenetic modification. In fact, conventional sequencing using bisulfite conversion could not distinguish 5hmC from 5mC. To overcome this limitation, recent advanced technologies using high-throughput sequencing enabled researchers to map 5hmC genome-wide [reviewed in Zhang et al. (2023)]. The prevailing view is that DNA methylation generally suppresses, whereas DNA hydroxymethylation promotes gene expression; however, the role of 5hmC at promoters, exons, introns, and gene bodies remains incompletely elucidated. As different brain regions have region-specific functions, TETs/global 5hmC expression and 5hmC at gene promoters in each brain region remain unknown. Studies have shown that 5hmC is linked to GABA signaling, drug addiction, alcohol drinking, and cognitive function in the AUD brain, and progression of HCC in alcoholic liver disease. Although previous studies have shown 5hmC in specific brain regions, the limited sample size and post-mortem or comorbid effects warrant the findings to be validated in animal models or large cohorts. Furthermore, a majority of the studies included only brains from male individuals, which could introduce sex-based effects to the findings. Therefore, future studies should aim to address these gaps using recent technologies to elucidate the overall picture of the hydroxymethylome in the brain and liver of AUD. Collectively, such a finding would contribute to the development of 5hmC as a biomarker and as an epigenetic target for therapeutic exploration in AUD treatment.

## References

[ref1] Agnihotri S. Wolf A. Munoz D. M. Smith C. J. Gajadhar A. Restrepo A. . (2011). A GATA4-regulated tumor suppressor network represses formation of malignant human astrocytomas. J. Exp. Med. 208, 689–702. doi: 10.1084/jem.20102099, 21464220 PMC3135351

[ref2] Ajoolabady A. Aslkhodapasandhokmabad H. Zhou Y. Ren J. (2022). Epigenetic modification in alcohol-related liver diseases. Med. Res. Rev. 42, 1463–1491. doi: 10.1002/med.21881, 35187693

[ref3] Andrade-Brito D. E. Núñez-Ríos D. L. Martínez-Magaña J. J. Nagamatsu S. T. Rompala G. Zillich L. . (2024). Neuronal-specific methylome and hydroxymethylome analysis reveal significant loci associated with alcohol use disorder. Front. Genet. 15:1345410. doi: 10.3389/fgene.2024.1345410, 38633406 PMC11021708

[ref4] Antunes C. Da Silva J. D. Guerra-Gomes S. Alves N. D. Ferreira F. Loureiro-Campos E. . (2021). Tet3 ablation in adult brain neurons increases anxiety-like behavior and regulates cognitive function in mice. Mol. Psychiatry 26, 1445–1457. doi: 10.1038/s41380-020-0695-7, 32103150

[ref5] Arvinden V. R. Deva Magendhra Rao A. K. Rajkumar T. Mani S. (2017). Regulation and functional significance of 5-hydroxymethylcytosine in cancer. Epigenomes 1:19. doi: 10.3390/epigenomes1030019

[ref6] Barbier E. Tapocik J. D. Juergens N. Pitcairn C. Borich A. Schank J. R. . (2015). DNA methylation in the medial prefrontal cortex regulates alcohol-induced behavior and plasticity. J. Neurosci. 35, 6153–6164. doi: 10.1523/JNEUROSCI.4571-14.2015, 25878287 PMC4397609

[ref7] Ben-Shachar S. Chahrour M. Thaller C. Shaw C. A. Zoghbi H. Y. (2009). Mouse models of MeCP2 disorders share gene expression changes in the cerebellum and hypothalamus. Hum. Mol. Genet. 18, 2431–2442. doi: 10.1093/hmg/ddp181, 19369296 PMC2694691

[ref8] Binder E. B. (2009). The role of FKBP5, a co-chaperone of the glucocorticoid receptor in the pathogenesis and therapy of affective and anxiety disorders. Psychoneuroendocrinology 34, S186–S195. doi: 10.1016/j.psyneuen.2009.05.021, 19560279

[ref9] Blaschke K. Ebata K. T. Karimi M. M. Zepeda-Martínez J. A. Goyal P. Mahapatra S. . (2013). Vitamin C induces Tet-dependent DNA demethylation and a blastocyst-like state in ES cells. Nature 500, 222–226. doi: 10.1038/nature12362, 23812591 PMC3893718

[ref10] Brabson J. P. Leesang T. Mohammad S. Cimmino L. (2021). Epigenetic regulation of genomic stability by vitamin C. Front. Genet. 12:675780. doi: 10.3389/fgene.2021.675780, 34017357 PMC8129186

[ref11] Brahadeeswaran S. Dasgupta T. Manickam V. Saraswathi V. Tamizhselvi R. (2023). NLRP3: a new therapeutic target in alcoholic liver disease. Front. Immunol. 14:1215333. doi: 10.3389/fimmu.2023.1215333, 37520548 PMC10374212

[ref12] Carrillo-Jimenez A. Deniz Ö. Niklison-Chirou M. V. Ruiz R. Bezerra-Salomão K. Stratoulias V. . (2019). TET2 regulates the neuroinflammatory response in microglia. Cell Rep. 29, 697–713.e8. doi: 10.1016/j.celrep.2019.09.013 31618637

[ref13] Chahrour M. Jung S. Y. Shaw C. Zhou X. Wong S. T. C. Qin J. . (2008). MeCP2, a key contributor to neurological disease, activates and represses transcription. Science 320, 1224–1229. doi: 10.1126/science.1153252, 18511691 PMC2443785

[ref14] Chen D. Yan Y. Wang X. Li S. Liu Y. Yu D. . (2021). Chronic alcohol exposure promotes HCC stemness and metastasis through β-catenin/miR-22-3p/TET2 axis. Aging 13, 14433–14455. doi: 10.18632/aging.203059, 34019487 PMC8202861

[ref15] Clark S. L. Chan R. F. Zhao M. Xie L. Y. Copeland W. E. Penninx B. W. J. H. . (2022). Dual methylation and hydroxymethylation study of alcohol use disorder. Addict. Biol. 27:e13114. doi: 10.1111/adb.13114, 34791764 PMC8891051

[ref16] Cruz B. Vozella V. Carper B. A. Xu J. C. Kirson D. Hirsch S. . (2023). FKBP5 inhibitors modulate alcohol drinking and trauma-related behaviors in a model of comorbid post-traumatic stress and alcohol use disorder. Neuropsychopharmacology 48, 1144–1154. doi: 10.1038/s41386-022-01497-w, 36396784 PMC10267127

[ref17] Dai W. Qiao X. Fang Y. Guo R. Bai P. Liu S. . (2024). Epigenetics-targeted drugs: current paradigms and future challenges. Signal Transduct. Target. Ther. 9:332. doi: 10.1038/s41392-024-02039-0, 39592582 PMC11627502

[ref18] Delatte B. Wang F. Ngoc L. V. Collignon E. Bonvin E. Deplus R. . (2016). Transcriptome-wide distribution and function of RNA hydroxymethylcytosine. Science 351, 282–285. doi: 10.1126/science.aac5253 26816380

[ref19] Dharavath R. N. Pina-Leblanc C. Tang V. M. Sloan M. E. Nikolova Y. S. Pangarov P. . (2023). GABAergic signaling in alcohol use disorder and withdrawal: pathological involvement and therapeutic potential. Front. Neural Circuits 17:1218737. doi: 10.3389/fncir.2023.1218737, 37929054 PMC10623140

[ref20] Diaz L. A. Winder G. S. Leggio L. Bajaj J. S. Bataller R. Arab J. P. (2025). New insights into the molecular basis of alcohol abstinence and relapse in alcohol-associated liver disease. Hepatology 82, 254–271. doi: 10.1097/HEP.0000000000000645 37862466

[ref21] Dick A. L. W. Zhao Q. Crossin R. Baker-Andresen D. Li X. Edson J. . (2021). Adolescent chronic intermittent toluene inhalation dynamically regulates the transcriptome and neuronal methylome within the rat medial prefrontal cortex. Addict. Biol. 26:e12937. doi: 10.1111/adb.12937 32638524

[ref22] Edenberg H. J. Koller D. L. Xuei X. Wetherill L. McClintick J. N. Almasy L. . (2010). Genome-wide association study of alcohol dependence implicates a region on chromosome 11. Alcohol. Clin. Exp. Res. 34, 840–852. doi: 10.1111/j.1530-0277.2010.01156.x 20201924 PMC2884073

[ref23] Ehrlich M. Ehrlich K. C. (2014). DNA cytosine methylation and hydroxymethylation at the borders. Epigenomics 6, 563–566. doi: 10.2217/epi.14.48, 25531248 PMC4321890

[ref24] Farokhnia M. Rentsch C. T. Chuong V. McGinn M. A. Elvig S. K. Douglass E. A. . (2022). Spironolactone as a potential new pharmacotherapy for alcohol use disorder: convergent evidence from rodent and human studies. Mol. Psychiatry 27, 4642–4652. doi: 10.1038/s41380-022-01736-y, 36123420 PMC10231646

[ref25] Feng L.-L. Liu R.-Y. An K. Tang S. Wu J. Yang Q. (2023). TET3 as a non-invasive screening tool for the detection of fibrosis in patients with chronic liver disease. Sci. Rep. 13:6382. doi: 10.1038/s41598-023-33564-7, 37076545 PMC10115894

[ref26] Finegersh A. Ferguson C. Maxwell S. Mazariegos D. Farrell D. Homanics G. E. (2015). Repeated vapor ethanol exposure induces transient histone modifications in the brain that are modified by genotype and brain region. Front. Mol. Neurosci. 8:39. doi: 10.3389/fnmol.2015.00039, 26300722 PMC4524924

[ref27] Fish K. N. Joffe M. E. (2022). Targeting prefrontal cortex GABAergic microcircuits for the treatment of alcohol use disorder. Front. Synaptic Neurosci. 14:936911. doi: 10.3389/fnsyn.2022.936911, 36105666 PMC9465392

[ref28] Friske M. M. Torrico E. C. Haas M. J. W. Borruto A. M. Giannone F. Hade A.-C. . (2025). A systematic review and meta-analysis on the transcriptomic signatures in alcohol use disorder. Mol. Psychiatry 30, 310–326. doi: 10.1038/s41380-024-02719-x, 39242950 PMC11649567

[ref29] Gao J. Liu B. Chen H. Xu P. Guo X. Yao D. . (2026). The role of DNA methylation in alcohol-mediated neurodevelopmental toxicity. Toxicology 519:154315. doi: 10.1016/j.tox.2025.154315, 41110586

[ref30] Gatta E. Auta J. Gavin D. P. Bhaumik D. K. Grayson D. R. Pandey S. C. . (2017). Emerging role of one-carbon metabolism and DNA methylation enrichment on δ-containing GABAA receptor expression in the cerebellum of subjects with alcohol use disorders (AUD). Int. J. Neuropsychopharmacol. 20, 1013–1026. doi: 10.1093/ijnp/pyx075, 29020412 PMC5716183

[ref31] Gatta E. Grayson D. R. Auta J. Saudagar V. Dong E. Chen Y. . (2021). Genome-wide methylation in alcohol use disorder subjects: implications for an epigenetic regulation of the cortico-limbic glucocorticoid receptors (NR3C1). Mol. Psychiatry 26, 1029–1041. doi: 10.1038/s41380-019-0449-6, 31239533 PMC6930366

[ref32] Gavin D. P. Kusumo H. Zhang H. Guidotti A. Pandey S. C. (2016). Role of growth arrest and DNA damage-inducible, beta in alcohol-drinking behaviors. Alcohol. Clin. Exp. Res. 40, 263–272. doi: 10.1111/acer.12965, 26842245 PMC4743544

[ref33] Granchi J. Salameh B. Miller B. Scaduto P. Russell W. K. Meyer T. D. . (2026). Multi-omics and electrophysiological examination of GABAA receptors in the dorsolateral prefrontal cortex of humans with alcohol use disorder. Mol. Psychiatry. doi: 10.1038/s41380-026-03598-0 PMC13441996 41965895

[ref34] Greer C. B. Wright J. Weiss J. D. Lazarenko R. M. Moran S. P. Zhu J. . (2021). Tet1 isoforms differentially regulate gene expression, synaptic transmission, and memory in the mammalian brain. J. Neurosci. 41, 578–593. doi: 10.1523/JNEUROSCI.1821-20.2020, 33262245 PMC7842754

[ref35] Hade A.-C. Philips M.-A. Reimann E. Jagomäe T. Eskla K.-L. Traks T. . (2021). Chronic alcohol use induces molecular genetic changes in the dorsomedial thalamus of people with alcohol-related disorders. Brain Sci. 11:435. doi: 10.3390/brainsci11040435, 33805312 PMC8066746

[ref36] Huggett S. B. Selveraj S. McGeary J. E. Ikeda A. S. Yuan E. Loeffel L. B. . (2026). Bulk and single-cell transcriptomic brain data identify overlapping processes and cell-types with human AUD and mammalian models of alcohol use. Transl. Psychiatry 16:212. doi: 10.1038/s41398-026-03919-5, 41896532 PMC13039797

[ref37] Inoue A. Zhang Y. (2011). Replication-dependent loss of 5-hydroxymethylcytosine in mouse preimplantation embryos. Science 334, 194–194. doi: 10.1126/science.1212483, 21940858 PMC3799877

[ref38] Jarczak J. Miszczak M. Radwanska K. (2023). Is DNA methylation in the brain a mechanism of alcohol use disorder? Front. Behav. Neurosci. 17:957203. doi: 10.3389/fnbeh.2023.957203, 36778133 PMC9908583

[ref39] Ji C. Nagaoka K. Zou J. Casulli S. Lu S. Cao K. Y. . (2019). Chronic ethanol-mediated hepatocyte apoptosis links to decreased TET1 and 5-hydroxymethylcytosine formation. FASEB J. 33, 1824–1835. doi: 10.1096/fj.201800736R, 30188753 PMC6338639

[ref40] Kamal H. Tan G. C. Ibrahim S. F. Shaikh M. F. Mohamed I. N. Mohamed R. M. P. . (2020). Alcohol use disorder, neurodegeneration, Alzheimer’s and Parkinson’s disease: interplay between oxidative stress, neuroimmune response and excitotoxicity. Front. Cell. Neurosci. 14:282. doi: 10.3389/fncel.2020.00282, 33061892 PMC7488355

[ref9001] Kang J. Park S. H. Khanam M. Park S. B. Shin S. Seo W. (2025). Impact of binge drinking on alcoholic liver disease. Arch. Pharm. Res 48, 212–223. 40035998 10.1007/s12272-025-01537-1

[ref41] Kang J. Park H. Kim E. (2016). IRSp53/BAIAP2 in dendritic spine development, NMDA receptor regulation, and psychiatric disorders. Neuropharmacology 100, 27–39. doi: 10.1016/j.neuropharm.2015.06.019 26275848

[ref42] Karpyak V. M. Winham S. J. Biernacka J. M. Cunningham J. M. Lewis K. A. Geske J. R. . (2014). Association of GATA4 sequence variation with alcohol dependence. Addict. Biol. 19, 312–315. doi: 10.1111/j.1369-1600.2012.00482.x, 22862823 PMC3504631

[ref43] Kharat S. S. Sharan S. K. (2025). 5-Hydroxymethylcytosine: a key epigenetic mark in cancer and chemotherapy response. Epigenetics Chromatin 18:73. doi: 10.1186/s13072-025-00636-z, 41243111 PMC12621374

[ref44] Khare T. Pai S. Koncevicius K. Pal M. Kriukiene E. Liutkeviciute Z. . (2012). 5-hmC in the brain is abundant in synaptic genes and shows differences at the exon-intron boundary. Nat. Struct. Mol. Biol. 19, 1037–1043. doi: 10.1038/nsmb.2372 22961382 PMC3465469

[ref46] Khom S. Rodriguez L. Gandhi P. Kirson D. Bajo M. Oleata C. S. . (2022). Alcohol dependence and withdrawal increase sensitivity of central amygdalar GABAergic synapses to the glucocorticoid receptor antagonist mifepristone in male rats. Neurobiol. Dis. 164:105610. doi: 10.1016/j.nbd.2022.105610, 34995754 PMC9301881

[ref47] Kissler J. L. Walker B. M. (2016). Dissociating motivational from physiological withdrawal in alcohol dependence: role of central amygdala κ-opioid receptors. Neuropsychopharmacology 41, 560–567. doi: 10.1038/npp.2015.183, 26105136 PMC5130131

[ref48] Koller G. Zill P. Soyka M. Adorjan K. Weiss C. Kern A. . (2019). Short-term changes in global methylation and hydroxymethylation during alcohol detoxification. Eur. Neuropsychopharmacol. 29, 897–903. doi: 10.1016/j.euroneuro.2019.05.001 31133368

[ref49] Koob G. F. Volkow N. D. (2016). Neurobiology of addiction: a neurocircuitry analysis. Lancet Psychiatry 3, 760–773. doi: 10.1016/S2215-0366(16)00104-8, 27475769 PMC6135092

[ref50] Kusumanchi P. Liang T. Zhang T. Ross R. A. Han S. Chandler K. . (2021). Stress-responsive gene FKBP5 mediates alcohol-induced liver injury through the hippo pathway and CXCL1 signaling. Hepatology 74:1234. doi: 10.1002/HEP.31800 33710653 PMC8435051

[ref51] Lai W. Zhang J. Sun J. Min T. Bai Y. He J. . (2024). Oxidative stress in alcoholic liver disease, focusing on proteins, nucleic acids, and lipids: a review. Int. J. Biol. Macromol. 278:134809. doi: 10.1016/j.ijbiomac.2024.134809 39154692

[ref52] Le Berre A. Fama R. Sullivan E. V. (2017). Executive functions, memory, and social cognitive deficits and recovery in chronic alcoholism: a critical review to inform future research. Alcohol. Clin. Exp. Res. 41, 1432–1443. doi: 10.1111/acer.13431, 28618018 PMC5531758

[ref53] Le T. N. Zhou Q.-P. Cobos I. Zhang S. Zagozewski J. Japoni S. . (2017). GABAergic interneuron differentiation in the basal forebrain is mediated through direct regulation of glutamic acid decarboxylase isoforms by dlx homeobox transcription factors. J. Neurosci. 37, 8816–8829. doi: 10.1523/JNEUROSCI.2125-16.2017, 28821666 PMC6596671

[ref54] Li Q. Pan Y. Zhang J. Hu B. Qin D. Liu S. . (2024). TET2 regulation of alcoholic fatty liver via Srebp1 mRNA in paraspeckles. IScience 27:109278. doi: 10.1016/j.isci.2024.109278, 38482502 PMC10933471

[ref55] Lindholm S. Werme M. Brené S. Franck J. (2001). The selective κ-opioid receptor agonist U50,488H attenuates voluntary ethanol intake in the rat. Behav. Brain Res. 120, 137–146. doi: 10.1016/S0166-4328(00)00368-5 11182162

[ref56] Lio C.-W. J. Rao A. (2019). TET enzymes and 5hmC in adaptive and innate immune systems. Front. Immunol. 10:210. doi: 10.3389/fimmu.2019.00210, 30809228 PMC6379312

[ref57] Liu J.-W. Zhang Z.-Q. Zhu Z.-C. Li K. Xu Q. Zhang J. . (2024). Loss of TET activity in the postnatal mouse brain perturbs synaptic gene expression and impairs cognitive function. Neurosci. Bull. 40, 1699–1712. doi: 10.1007/s12264-024-01302-2, 39395911 PMC11607366

[ref58] Logrip M. L. Janak P. H. Ron D. (2008). Dynorphin is a downstream effector of striatal BDNF regulation of ethanol intake. FASEB J. 22, 2393–2404. doi: 10.1096/fj.07-099135 18310464

[ref59] Lohoff F. W. Clarke T.-K. Kaminsky Z. A. Walker R. M. Bermingham M. L. Jung J. . (2022). Epigenome-wide association study of alcohol consumption in N=8161 individuals and relevance to alcohol use disorder pathophysiology: identification of the cystine/glutamate transporter SLC7A11 as a top target. Mol. Psychiatry 27, 1754–1764. doi: 10.1038/s41380-021-01378-6, 34857913 PMC9095480

[ref60] López-Moyado I. F. Ko M. Hogan P. G. Rao A. (2024). TET enzymes in the immune system: from DNA demethylation to immunotherapy, inflammation, and cancer. Annu. Rev. Immunol. 42, 455–488. doi: 10.1146/annurev-immunol-080223-044610, 38360546

[ref61] Lussier A. A. Stepien K. A. Neumann S. M. Pavlidis P. Kobor M. S. Weinberg J. (2015). Prenatal alcohol exposure alters steady-state and activated gene expression in the adult rat brain. Alcohol. Clin. Exp. Res. 39, 251–261. doi: 10.1111/acer.12622, 25684047 PMC4833439

[ref62] Madugundu G. S. Cadet J. Wagner J. R. (2014). Hydroxyl-radical-induced oxidation of 5-methylcytosine in isolated and cellular DNA. Nucleic Acids Res. 42, 7450–7460. doi: 10.1093/nar/gku334, 24852253 PMC4066766

[ref63] Marshall L. L. Killinger B. A. Ensink E. Li P. Li K. X. Cui W. . (2020). Epigenomic analysis of Parkinson’s disease neurons identifies Tet2 loss as neuroprotective. Nat. Neurosci. 23, 1203–1214. doi: 10.1038/s41593-020-0690-y 32807949

[ref64] Maunakea A. K. Chepelev I. Cui K. Zhao K. (2013). Intragenic DNA methylation modulates alternative splicing by recruiting MeCP2 to promote exon recognition. Cell Res. 23, 1256–1269. doi: 10.1038/cr.2013.110, 23938295 PMC3817542

[ref65] Mauro K. L. Helton S. G. Rosoff D. B. Luo A. Schwandt M. Jung J. . (2018). Association analysis between genetic variation in GATA binding protein 4 (GATA4) and alcohol use disorder. Alcohol Alcohol. 53, 361–367. doi: 10.1093/alcalc/agx120, 29415147 PMC6016699

[ref66] McKinney B. Ding Y. Lewis D. A. Sweet R. A. (2017). DNA methylation as a putative mechanism for reduced dendritic spine density in the superior temporal gyrus of subjects with schizophrenia. Transl. Psychiatry 7, e1032–e1032. doi: 10.1038/tp.2016.297, 28195572 PMC5438028

[ref67] Mihalek R. M. Bowers B. J. Wehner J. M. Kralic J. E. VanDoren M. J. Morrow A. L. . (2001). GABA(a)-receptor delta subunit knockout mice have multiple defects in behavioral responses to ethanol. Alcohol. Clin. Exp. Res. 25, 1708–1718. doi: 10.1111/j.1530-0277.2001.tb02179.x 11781502

[ref68] Mikkelsen A. C. D. Kjærgaard K. Schapira A. H. V. Mookerjee R. P. Thomsen K. L. (2025). The liver–brain axis in metabolic dysfunction-associated steatotic liver disease. Lancet Gastroenterol. Hepatol. 10, 248–258. doi: 10.1016/S2468-1253(24)00320-0, 39701123

[ref69] Nie H. Rewal M. Gill T. M. Ron D. Janak P. H. (2011). Extrasynaptic δ-containing GABA _A_ receptors in the nucleus accumbens dorsomedial shell contribute to alcohol intake. Proc. Natl. Acad. Sci. USA 108, 4459–4464. doi: 10.1073/pnas.1016156108, 21368141 PMC3060220

[ref70] Niinep K. Anier K. Eteläinen T. Piepponen P. Kalda A. (2021). Repeated ethanol exposure alters DNA methylation status and dynorphin/kappa-opioid receptor expression in nucleus accumbens of alcohol-preferring AA rats. Front. Genet. 12:750142. doi: 10.3389/fgene.2021.750142, 34899839 PMC8652212

[ref71] Occean J. R. Yang N. Sun Y. Dawkins M. S. Munk R. Belair C. . (2024). Gene body DNA hydroxymethylation restricts the magnitude of transcriptional changes during aging. Nat. Commun. 15:6357. doi: 10.1038/s41467-024-50725-y 39069555 PMC11284234

[ref72] Parker M. J. Weigele P. R. Saleh L. (2019). Insights into the biochemistry, evolution, and biotechnological applications of the ten-eleven translocation (TET) enzymes. Biochemistry 58, 450–467. doi: 10.1021/acs.biochem.8b01185 30571101

[ref73] Pirola C. J. Scian R. Gianotti T. F. Dopazo H. Rohr C. Martino J. S. . (2015). Epigenetic modifications in the biology of nonalcoholic fatty liver disease. Medicine 94:e1480. doi: 10.1097/MD.0000000000001480 26356709 PMC4616643

[ref74] Pla R. Stanco A. Howard M. A. Rubin A. N. Vogt D. Mortimer N. . (2018). Dlx1 and Dlx2 promote interneuron GABA synthesis, synaptogenesis, and dendritogenesis. Cereb. Cortex 28, 3797–3815. doi: 10.1093/cercor/bhx241, 29028947 PMC6188538

[ref75] Pratt K. J. B. Shea J. M. Remesal-Gomez L. Bieri G. Smith L. K. Couthouis J. . (2022). Loss of neuronal Tet2 enhances hippocampal-dependent cognitive function. Cell Rep. 41:111612. doi: 10.1016/j.celrep.2022.111612, 36351399 PMC10032941

[ref76] Qiu J. Wu S. Wang P. Zhou Y. Wang Z. Sun Y. . (2023). MiR-488-5p mitigates hepatic stellate cell activation and hepatic fibrosis via suppressing TET3 expression. Hepatol. Int. 17, 463–475. doi: 10.1007/s12072-022-10404-w, 36001230 PMC10119239

[ref77] Rehm J. (2011). The risks associated with alcohol use and alcoholism. Alcohol Res. Health 34, 135–143. 22330211 PMC3307043

[ref78] Routh S. Manickam V. (2024). Epigenetic alterations dictating the inflammation: a view through pancreatitis. Life Sci. 338:122383. doi: 10.1016/j.lfs.2023.122383, 38158041

[ref79] Rudenko A. Dawlaty M. M. Seo J. Cheng A. W. Meng J. Le T. . (2013). Tet1 is critical for neuronal activity-regulated gene expression and memory extinction. Neuron 79, 1109–1122. doi: 10.1016/j.neuron.2013.08.003, 24050401 PMC4543319

[ref80] Sakharkar A. J. Kyzar E. J. Gavin D. P. Zhang H. Chen Y. Krishnan H. R. . (2019). Altered amygdala DNA methylation mechanisms after adolescent alcohol exposure contribute to adult anxiety and alcohol drinking. Neuropharmacology 157:107679. doi: 10.1016/j.neuropharm.2019.107679, 31229451 PMC6681823

[ref81] Sakharkar A. J. Tang L. Zhang H. Chen Y. Grayson D. R. Pandey S. C. (2014). Effects of acute ethanol exposure on anxiety measures and epigenetic modifiers in the extended amygdala of adolescent rats. Int. J. Neuropsychopharmacol. 17, 2057–2067. doi: 10.1017/S1461145714001047, 24968059 PMC4213292

[ref82] Siciliano C. A. Calipari E. S. Cuzon Carlson V. C. Helms C. M. Lovinger D. M. Grant K. A. . (2015). Voluntary ethanol intake predicts κ-opioid receptor supersensitivity and regionally distinct dopaminergic adaptations in macaques. J. Neurosci. 35, 5959–5968. doi: 10.1523/JNEUROSCI.4820-14.2015, 25878269 PMC4397597

[ref83] Sirohi S. Bakalkin G. Walker B. M. (2012). Alcohol-induced plasticity in the dynorphin/kappa-opioid receptor system. Front. Mol. Neurosci. 5:95. doi: 10.3389/fnmol.2012.00095, 23060746 PMC3459013

[ref84] Szulwach K. E. Li X. Li Y. Song C.-X. Wu H. Dai Q. . (2011). 5-hmC–mediated epigenetic dynamics during postnatal neurodevelopment and aging. Nat. Neurosci. 14, 1607–1616. doi: 10.1038/nn.2959, 22037496 PMC3292193

[ref85] Tammen S. A. Park J. E. Shin P. K. Friso S. Chung J. Choi S.-W. (2016). Iron supplementation reverses the reduction of hydroxymethylcytosine in hepatic DNA associated with chronic alcohol consumption in rats. J. Cancer Prev. 21, 264–270. doi: 10.15430/JCP.2016.21.4.264, 28053961 PMC5207611

[ref86] Tang X. Wu L. Zhu J. Xu M. Li S. Zeng G. . (2023). GABAergic neurons differentiated from BDNF- and Dlx2-modified neural stem cells restore disrupted neural circuits in brainstem stroke. Stem Cell Res. Ther. 14:170. doi: 10.1186/s13287-023-03378-5, 37365654 PMC10294474

[ref87] Traube F. R. Carell T. (2017). The chemistries and consequences of DNA and RNA methylation and demethylation. RNA Biol. 14, 1099–1107. doi: 10.1080/15476286.2017.1318241, 28440690 PMC5699545

[ref88] Tsermpini E. E. Plemenitaš Ilješ A. Dolžan V. (2022). Alcohol-induced oxidative stress and the role of antioxidants in alcohol use disorder: a systematic review. Antioxidants 11:1374. doi: 10.3390/antiox11071374, 35883865 PMC9311529

[ref89] Underwood M. D. Bakalian M. J. Dwork A. J. Min E. Mann J. J. Arango V. (2019). GAD mRNA in orbital prefrontal cortex and anterior cingulate cortex in alcoholics compared with nonpsychiatric controls: a negative postmortem study. J. Psychiatry Brain Sci. 4:e190007. doi: 10.20900/jpbs.20190007 PMC6594560 31245628

[ref90] Warden A. S. Salem N. A. Brenner E. Sutherland G. T. Stevens J. Kapoor M. . (2026). Integrative genomics approach identifies glial transcriptomic dysregulation and risk in the cortex of individuals with alcohol use disorder. Biol. Psychiatry 99, 34–48. doi: 10.1016/j.biopsych.2025.02.895, 40024496 PMC12667600

[ref91] Wee S. Koob G. F. (2010). The role of the dynorphin–κ opioid system in the reinforcing effects of drugs of abuse. Psychopharmacology 210, 121–135. doi: 10.1007/s00213-010-1825-8, 20352414 PMC2879894

[ref92] Wei H. Yu C. Zhang C. Ren Y. Guo L. Wang T. . (2023). Butyrate ameliorates chronic alcoholic central nervous damage by suppressing microglia-mediated neuroinflammation and modulating the microbiome-gut-brain axis. Biomed. Pharmacother. 160:114308. doi: 10.1016/j.biopha.2023.114308, 36709599

[ref93] Wen L. Li X. Yan L. Tan Y. Li R. Zhao Y. . (2014). Whole-genome analysis of 5-hydroxymethylcytosine and 5-methylcytosine at base resolution in the human brain. Genome Biol. 15:R49. doi: 10.1186/gb-2014-15-3-r49 24594098 PMC4053808

[ref94] Willis C. White J. D. Minto M. S. Quach B. C. Han S. Tao R. . (2025). Gene expression differences associated with alcohol use disorder in human brain. Mol. Psychiatry 30, 1617–1626. doi: 10.1038/s41380-024-02777-1, 39394458 PMC11919698

[ref95] Yu H. Su Y. Shin J. Zhong C. Guo J. U. Weng Y.-L. . (2015). Tet3 regulates synaptic transmission and homeostatic plasticity via DNA oxidation and repair. Nat. Neurosci. 18, 836–843. doi: 10.1038/nn.4008, 25915473 PMC4446239

[ref96] Zannas A. S. Chrousos G. P. (2017). Epigenetic programming by stress and glucocorticoids along the human lifespan. Mol. Psychiatry 22, 640–646. doi: 10.1038/mp.2017.35, 28289275

[ref97] Zhang X. Zhang Y. Wang C. Wang X. (2023). TET (ten-eleven translocation) family proteins: structure, biological functions and applications. Signal Transduct. Target. Ther. 8:297. doi: 10.1038/s41392-023-01537-x, 37563110 PMC10415333

[ref98] Zhao J. Gu T. Gao C. Miao G. Palma-Gudiel H. Yu L. . (2025). Brain 5-hydroxymethylcytosine alterations are associated with Alzheimer’s disease neuropathology. Nat. Commun. 16:2842. doi: 10.1038/s41467-025-58159-w, 40121201 PMC11929800

[ref99] Zhong L. Zhu J. Lv T. Chen G. Sun H. Yang X. . (2010). Ethanol and its metabolites induce histone lysine 9 acetylation and an alteration of the expression of heart development-related genes in cardiac progenitor cells. Cardiovasc. Toxicol. 10, 268–274. doi: 10.1007/s12012-010-9081-z, 20811785

[ref100] Zhubi A. Chen Y. Dong E. Cook E. H. Guidotti A. Grayson D. R. (2014). Increased binding of MeCP2 to the GAD1 and RELN promoters may be mediated by an enrichment of 5-hmC in autism spectrum disorder (ASD) cerebellum. Transl. Psychiatry 4, e349–e349. doi: 10.1038/tp.2013.123, 24448211 PMC3905233

